# *In ovo* myo-inositol administration: impacts on growth performance and metabolic profiles in broiler chickens

**DOI:** 10.3389/fphys.2025.1706565

**Published:** 2026-02-09

**Authors:** Nataliia Shomina, Vera Sommerfeld, Markus Rodehutscord, Korinna Huber

**Affiliations:** Institute of Animal Science, University of Hohenheim, Stuttgart, Germany

**Keywords:** broiler chickens, *in ovo* injection, metabolic profiles, myo-inositol, performance

## Abstract

Myo-inositol (MI) plays key roles in cellular signaling, membrane structure, and metabolic regulation, with its effects in poultry primarily explored through direct dietary MI supplementation. In this study, we aimed to assess the effects of *in ovo* MI administration on post-hatch performance and metabolism of broiler chickens. A total of 480 fertilized Ross 308 eggs were divided into four groups and, on day 17 of incubation, were injected with 12 μmol/mL MI (MI 12), 24 μmol/mL MI (MI 24), 0.9% saline (positive control, PC), or left non-injected (negative control, NC). After hatching, broilers were group-housed in floor pens (8 pens per treatment), with 12 birds per pen, and fed a standard diet for 35 days. At d 35, one bird per pen was slaughtered, sex was identified, and blood and tissues were collected to assess MI concentrations, the expression of inositol monophosphatase 1 (IMPase 1) and myo-inositol oxygenase (MIOX), and plasma metabolite profiles. There was no adverse effect of MI *in ovo* administration on hatchability and body weight (BW) of hatchlings. During the growing period, BW was lower in MI-injected groups from day 14 onward, along with reduced average daily weight gain; however, no differences were observed in the feed conversion ratio. The survival rate was higher in MI-injected groups during days 0–21, with a positive trend until the end of the experiment. MI concentrations in plasma and tissues, along with the expression of IMPase 1 and MIOX, were not altered by treatment. Plasma metabolomics revealed higher C2 and C9 acylcarnitines, threonine, and sarcosine, along with lower serotonin, and notable changes in phosphatidylcholines and sphingolipids in MI-injected versus no-MI groups, potentially reflecting alterations in mitochondrial β-oxidation pathways, diacylglycerol-associated signaling, amino-acid-related metabolism, and peripheral serotonin metabolism. Sex-specific differences in plasma MI and metabolite profiles were detected, with male birds demonstrating reduced plasma MI concentrations, Fisher ratio, and carnosine levels, indicative of a metabolic state possibly associated with higher anabolic pressure or subclinical inflammatory activation. These findings highlight the potential of *in ovo* MI administration to induce subtle but persistent metabolic reprogramming and underscore the need for further studies to clarify its long-term consequences for metabolic resilience and performance in both sexes.

## 1 Introduction

Myo-inositol (MI), a stereoisomer of inositol (cyclohexane-1,2,3,4,5,6-hexol), is a sugar cyclic polyalcohol that plays essential roles in eukaryotes. It serves as a structural component of inositol phosphates (InsPs), which act as second messengers in intracellular signaling, and phosphoinositides (PIPs), involved in lipid signaling, cell communication, and membrane trafficking (Croze and Soulage, 2013). MI is obtained from dietary sources and endogenous body synthesis and is considered semi-essential as its availability may be limited under certain physiological or pathological conditions (Huber, 2016; Gonzalez-Uarquin et al., 2020c).

Endogenously, MI is synthesized *de novo* from D-glucose or regenerated from PIP and InsP. The final step of its synthesis is catalyzed by inositol monophosphatase 1 (IMPase 1), which dephosphorylates inositol monophosphate (InsP_1_) to release free MI for subsequent use in the phosphatidylinositol (PI) synthesis pathway. MI catabolism occurs in the kidney, where myo-inositol oxygenase (MIOX) converts MI to D-glucuronic acid (Croze and Soulage, 2013). Thus, the expression patterns of IMPase and MIOX indicate an intrinsic capacity to sustain MI homeostasis via its regeneration and catabolic conversion processes.

In poultry nutrition, strategies to complement or enhance the MI pool primarily focus on dietary interventions, such as direct MI supplementation or stimulation of MI release from phytate via dietary phytases (Gonzalez-Uarquin et al., 2020a; Rodehutscord et al., 2022). Early studies provided evidence for the metabolic importance of MI in broiler chickens: Zyła et al. (2004) and Zyła et al. (2013) reported that dietary MI supplementation improved body weight (BW), feed intake, and feed conversion rate (FCR). Subsequent studies (Pirgozliev et al., 2019; Gonzalez Uarquin et al., 2020a; Sommerfeld et al., 2018a) demonstrated only minor or no effects of MI-enriched diets on broiler growth performance, suggesting that its efficacy may be limited or condition-specific. Dietary MI supplementation consistently increased MI concentrations in the small intestine and blood plasma of broiler chickens (Sommerfeld et al., 2018a; Pirgozliev et al., 2019), indicating enhanced intestinal absorption. However, increased ileal MI concentrations were not accompanied by changes in the liver or kidney MI content (Sprigg et al., 2022). Although dietary MI supplementation increased plasma MI concentrations in both broilers and laying hens, it affected the plasma metabolite profile differently. In broilers, MI supplementation led to significant increases in serotonin and dopamine levels (Gonzalez-Uarquin et al., 2020a). In laying hens, only minor changes were detected in plasma metabolites, including lower methionine sulfoxide and kynurenine concentrations and higher symmetric dimethylarginine concentrations, suggesting weak anti-inflammatory and antioxidative effects of MI (Szentgyörgyi et al., 2025). Although increased intestinal and plasma MI levels in laying hens were associated with higher MI deposition in the egg yolk, the overall transfer to the egg remained limited, suggesting that *de novo* synthesis is the primary contributor to the egg MI content (Sommerfeld et al., 2025).

Given the established biological functions of MI and its transfer into the egg, it is plausible that MI could play an important role in embryo development, although specific studies in poultry are lacking. Research in other farm animals demonstrated that supplementation of MI to culture media during the embryo preimplantation stage enhanced blastocyst formation, expansion, and hatching rates in rabbits and bovines, ultimately supporting the development of healthy offspring (Holm et al., 1999; Warner et al., 2003). MI was also shown to possess antioxidant properties, mitigating oxidative stress particularly during embryo culture by activating cellular defense pathways (Jiang et al., 2013; Ali et al., 2024). In poultry, phytase supplementation (500–4500 FTU/kg) in breeder diets increased the MI content in the yolk sac of hatchlings, reduced late embryo mortality, and improved performance outcomes by enhancing chick growth and feed efficiency up to 21 days of age, which was likely associated with systemic MI influence (Granghelli et al., 2023).

Considering the limited natural hen-to-egg transfer of MI and its proposed roles in supporting embryonic development, targeted *in ovo* supplementation offers a controlled means to elevate MI availability during a critical developmental window. Numerous studies have demonstrated that *in ovo* nutrient delivery can induce lasting modifications in intestinal development, immune competence, and systemic metabolic regulation in poultry species (Das et al., 2021; Dunislawska et al., 2021). Among the available injection sites, the amniotic cavity is particularly effective at late incubation stages as substances delivered into this compartment are ingested and get deposited in the lungs and intestine due to the rhythmic respiratory movements of the late-term embryo (Jochemsen and Jeurissen, 2002; Uni et al., 2005). Once swallowed, MI would be expected to pass through the gastrointestinal tract with subsequent absorption into circulation, potentially leading to a transient increase in systemic and intracellular MI availability. Such an increase may interact with MI-regulated pathways involved in phosphoinositide signaling, glucose metabolism, mitochondrial function, and oxidative stress responses, influencing tissue maturation, cellular energy set-points, and postnatal metabolic trajectories. Based on this rationale, the aim of the present study was to transiently increase MI availability during late embryogenesis of broiler chickens through *in ovo* injection and to assess its consequences for postnatal metabolic regulation. We evaluated outcomes after an extended grow-out period (35 days), a phase characterized by high metabolic load, rapid muscle accretion, oxidative demands, and active lipid mobilization, to determine whether MI-induced developmental adaptations resulted in persistent changes in the metabolic phenotype, consistent with the concept of metabolic imprinting.

## 2 Materials and methods

This study was part of the interdisciplinary Research Unit P-Fowl–Inositol phosphates and myo-inositol in the domestic fowl: Exploring the interface of genetics, physiology, microbiome, and nutrition (https://p-fowl.uni-hohenheim.de/). The animal trial was conducted at the Agricultural Experiment Station of the University of Hohenheim, Germany. It was approved by the Regierungspräsidium Tübingen, Germany (approval No. HOH 75/24-460a) and conducted in accordance with the German Animal Welfare Legislation.

### 2.1 Eggs and incubation set up

A total of 550 fertilized broiler eggs (Ross 308 strain) were obtained from a single 52-week-old breeder flock in Baden-Württemberg, Germany. The eggs were weighed and distributed into four groups of 120 eggs each while establishing minimal variation in average egg weight across groups (67.3 ± 0.4 g). To minimize positional effects during incubation, each group was equally represented on two replicate incubation trays, with trays in both front and back positions within the incubator. The remaining eggs were incubated separately as a stock group, intended to replace non-fertile or early-dead embryos in the event of high losses. All eggs were individually marked with their respective group numbers and placed into the setter (Masalles 2600-I-HLC, Masalles S.L., Spain) equipped with an automatic turning system operating once per hour. Incubation was conducted under standard conditions: from day 1 to day 17, eggs were maintained at 37.8 °C with 65% relative humidity. After *in ovo* injection on day 17, the eggs were transferred to a hatcher (Masalles 5200-N-HLC, Masalles S.L., Spain) set at 37.5 °C and 73% relative humidity. Candling was performed on days 11 and 16 of incubation to identify and remove non-fertile eggs and those with dead embryos. After candling on day 16, the final experimental groups were established, each comprising 110 viable, fertilized eggs with live embryos.

### 2.2 Myo-inositol *in ovo* injection

MI (cell culture grade; cat. J62886.18, Thermo Fisher GmbH, Kandel, Germany) and 0.9% saline (cat. 13423, Honeywell Fluka™, Germany) solutions were prepared a day before the *in ovo* injection. To prepare the MI solutions, 0.9% saline was used as a solvent. All solutions were sterilized by autoclaving after preparation. The experimental design included four groups of eggs: (1) the MI 12 group received an *in ovo* injection of 1 mL solution containing 12 μmol/mL of MI; (2) the MI 24 group was injected with 1 mL of a solution containing 24 μmol/mL of MI; (3) the positive control (PC) group received an *in ovo* injection of 1 mL of 0.9% saline; (4) the negative control (NC) group served as a non-injected control and did not receive any injection. *In ovo* injections were performed into the amniotic fluid on day 17 of incubation (405–410 h post-setting) following the procedure described by Uni and Ferket (2003), with minor modifications. In brief, the eggshell surface was disinfected with 70% ethanol. A hole was made at the blunt end of the egg using a sterile 19G needle, and 1 mL of the respective solution was injected using a syringe fitted with a 23G needle. The needle was inserted to its full length (approximately 25 mm) to ensure delivery into the amniotic cavity. After injection, the shell surface was disinfected again with 70% ethanol, and the hole was sealed with melted paraffin wax. The total duration of egg handling outside the incubator did not exceed 20 min. For the NC group, eggs were also removed from the incubator and kept under the same conditions and for the same duration as the injected groups to account for handling effects. Following injection, eggs were placed into four sanitized hatching trays (one tray per group), and incubation continued in the hatcher.

### 2.3 Animals and housing

On the day of hatch, chicks were counted in each treatment group, and hatchability was calculated as the percentage of hatched chicks relative to the number of treated eggs per group. A total of 384 unsexed chicks (96 per group) started in the growth trial. After weighing in subgroups, the chicks were allocated to 32 floor pens (110 × 230 cm ground area, 200 cm height) with deep litter bedding, at a stocking density of 12 chicks per pen. Pens were assigned to the four treatment groups using a randomized complete block design, with eight replicate pens per group. Birds received a commercial starter diet (ME = 12.4 MJ/kg; CP = 21.5%; Landkornstarter, Deuka) from day 0 to day 14, followed by a grower diet (ME = 12.4 MJ/kg; CP = 20%; Landkornmast, Deuka) from day 14 to day 35. Feed and tap water were provided *ad libitum* throughout the trial. During the first 3 days after placement, the temperature in the animal house was maintained at 34 °C, and lighting was continuous. Thereafter, the temperature was gradually reduced by approximately 1 °C every 2 days until reaching 22 °C on day 28, which was maintained until the end of the experiment, whereas the lighting program was adjusted to a cycle of 18 h light (05:00–23:00 h) and 6 h dark (23:00–05:00 h). Animals were monitored twice daily, in the morning (around 08:00) and afternoon (around 16:00), and all mortalities were recorded. Chicks and feed were weighed weekly (days 7, 14, 21, 28, and 35) to calculate average BW, mortality-corrected average daily feed intake (ADFI), average daily gain (ADG), and FCR on a pen basis. The survival rate (SR) was determined weekly as the proportion of birds remaining alive at the end of each week, expressed as a percentage of the initial number of birds placed.

### 2.4 Slaughtering and sampling

The day before slaughter, birds were weighed individually, and one bird with a weight closest to the mean pen weight was selected for slaughter and marked with a color on the left wing. On the day of slaughter, the selected birds were weighed again and stunned using a gas mixture of 35% CO_2_, 35% N_2_, and 30% O_2_, followed by decapitation, and trunk blood was collected into EDTA tubes. To separate plasma, these tubes were centrifuged at 1,000 × *g* for 10 min at room temperature. The plasma was then aliquoted, shock-frozen in liquid nitrogen, and stored on dry ice until transfer to −80 °C for long-term storage. Following blood collection, birds were promptly eviscerated, and sex was determined post-slaughter. Using surgical instruments, the tissue samples were obtained from the right lobe of the liver, the left kidney, and the breast muscle (pectoralis major) on the left side of the keel bone. The samples were rinsed in ice-cold physiological saline, cut into small pieces, shock-frozen in liquid nitrogen, and placed in prechilled cryotubes. These tubes were kept on dry ice until transfer to storage at −80 °C.

### 2.5 Tissue grinding and homogenization

Liver, kidney, and breast muscle samples were ground under liquid nitrogen using a mortar and pestle. For homogenization, 420 mg of liver and kidney tissues or 140 mg of muscle tissues were transferred to lysing matrix tubes (MP Biomedicals, France) pre-filled with silica beads. For MI and MIOX determination, the samples were mixed with 500 µL of 1× phosphate-buffered saline (PBS) supplemented with a protease inhibitor cocktail (Complete Mini; Hoffmann-La Roche, Mannheim, Germany). To detect the IMPase 1 levels, liver samples were prepared using the same protocol, whereas muscle samples were diluted by mixing 140 mg of tissue with 750 µL of the same PBS/protease inhibitor solution. Homogenization was performed using a FastPrep-24 5G instrument (MP Biomedicals, Shanghai, China) at 6 m/s for 30 s, repeated three times for liver and kidney samples and four times for muscle samples, with 60 s cooling intervals on wet ice between cycles. The homogenates were then centrifuged at 1,500 × *g* for 15 min at 4 °C (Centrifuge 5424R; Eppendorf, Hamburg, Germany). The resulting supernatants were carefully collected, aliquoted, and stored at −80 °C until further analysis.

### 2.6 Protein quantification

For protein quantification, one aliquot of each liver and kidney homogenate was diluted in distilled water at a ratio of 1:500. Breast muscle homogenates were diluted at a ratio of either 1:400 or 1:200, depending on the respective tissue preparation protocol described above. Protein concentrations were determined using the Bradford assay (Bradford Reagent, 5×; SERVA, Heidelberg, Germany), following the manufacturer’s instructions. All protein measurements were conducted in triplicate.

### 2.7 Myo-inositol determination in egg components, plasma, and tissues

Before setting eggs for incubation, five eggs from the batch were opened, and the yolk and albumen were separated and weighed individually. Each component was then frozen, freeze-dried, and reweighed to determine dry mass. The dried samples were pulverized using a mortar and pestle and stored at −20 °C until further analysis. Pulverized yolk and albumen samples were analyzed for MI using gas chromatography–mass spectrometry (GC-MS) after chemical derivatization, as described by Sommerfeld et al. (2018b).

MI concentrations in plasma and tissue samples were quantified using a commercially available enzymatic assay kit (K-INOSL; Megazyme International, Ireland). The assay is based on the enzymatic oxidation of MI and the subsequent formation of a stable iodonitrotetrazolium chloride (INT)–formazan product, the absorbance of which at 492 nm is directly proportional to the MI concentration. Undiluted plasma samples were used for analysis, whereas initial tissue homogenates were diluted in distilled water before measurement (liver and kidney at 1:120; breast muscle at 1:50). The assay was performed on 96-well microtiter plates (655101; Greiner Bio-One GmbH, Frickenhausen, Germany), with eight samples analyzed per run. MI concentrations were calculated using a standard curve, with plasma results expressed in millimoles per liter (mmol/L) and tissue results expressed as milligrams per gram of protein (mg/g protein).

### 2.8 Inositol monophosphatase 1 and MIOX expressions

IMPase 1 expression in liver and breast muscles and MIOX expression in kidney samples were quantified using commercial enzyme-linked immunosorbent assay (ELISA) kits (Chicken IMPA1 ELISA Kit, MBS7235623; Chicken MIOX ELISA Kit, MBS7215577; MyBioSource, San Diego, CA). All measurements were performed according to the manufacturer’s instructions. The reported intra-assay and inter-assay coefficients of variation were 5.5% and 7.3%, respectively. For the assay, 100 µL of each tissue homogenate was used. Liver homogenates contained 69.8 ± 2.9 mg/mL protein, muscle homogenates contained 16.3 ± 0.9 mg/mL protein, and kidney homogenates contained 74.2 ± 1.9 mg/mL (mean ± SEM) protein. Each homogenate was mixed with the balance solution provided in the kit and incubated for 1 h at 37 °C. Liver and muscle samples were incubated with the horseradish peroxidase (HRP)-conjugated antibody specific for IMPase 1, whereas kidney samples were incubated with the HRP-conjugated antibody specific for MIOX. After incubation, the plates were washed manually five times and then incubated with the substrate for horseradish peroxidase. When the enzyme–substrate reaction produced a blue color, the stop solution was added to terminate the reaction, resulting in a yellow color. The intensity of the yellow color, measured spectrophotometrically at 450 nm using a microplate reader (Infinite M Nano, TECAN, Salzburg, Austria), was inversely proportional to the concentration of the target enzyme because both kits use a competitive ELISA format. Standard curves were generated by plotting absorbance values against known standard concentrations. Sample concentrations were then calculated using a five-parameter logistic curve fit (Magellan software, Tecan GmbH, 2016, Salzburg, Austria). All values were normalized to the protein concentration of the homogenate and expressed as picograms (pg) of enzyme/mg of the total protein.

### 2.9 Targeted metabolomics approach

Metabolomics analyses were performed by Biocrates Life Sciences AG (Innsbruck, Austria) using the AbsoluteIDQ™ p180 kit (Biocrates, Innsbruck, Austria). The kit enables the quantification of metabolites from the following classes: amino acids (AAs), acylcarnitines (ACs), biogenic amines, hexoses, lysophosphatidylcholines (LPCs), phosphatidylcholines (PCs), and sphingomyelins (SMs). The assay is based on phenyl isothiocyanate derivatization in the presence of internal standards, followed by flow injection analysis–tandem mass spectrometry (FIA-MS/MS) for ACs, LPCs, PCs, SMs, and hexoses, along with liquid chromatography–tandem mass spectrometry (LC-MS/MS) for AAs and biogenic amines. Measurements were conducted using a SCIEX 4000 QTRAP® (AB Sciex, Framingham, MA, United States) or a Xevo TQ-S Micro (Waters, Milford, MA, United States) instrument with electrospray ionization. The experimental metabolomics measurement technique is described in detail by patents EP1897014B1 and EP1875401B1. All measurements were performed according to the certified guidelines and protocols of Biocrates by applying validated analytical methods. Plasma metabolite concentrations were provided in micromoles per liter (µmol/L) by Biocrates as raw data.

### 2.10 Bioinformatics and statistical analyses

Statistical analyses were performed in SAS (version 9.3; SAS Institute Inc., Cary, NC, United States), using the MIXED procedure and pairwise *t*-tests for *post hoc* comparisons.

For variables calculated at the pen level (average BW, ADG, ADFI, FCR corrected for mortality, and SR), the pen was considered the experimental unit, and the following model was used:

$$Y_{\text{ijkl}}=\mu +\alpha_{i}+\gamma_{k}+\varepsilon_{\text{ijk}},$$

where Y_ijk_ is the response variable, *μ* is the overall mean, α_i_ is the effect of injection (fixed), γ_k_ is the effect of block (random), and ε_ijk_ is the residual error.

For variables calculated at the individual level (blood, liver, kidney, and breast muscle data), the bird was considered the experimental unit, and the following model was used:

$$Y_{\text{ijkl}}=\mu +\alpha_{i}+\beta_{j}+\gamma_{k}+\varepsilon_{\text{ijk}},$$

where Y_ijk_ is the response variable, *μ* is the overall mean, α_i_ is the effect of injection (fixed), β_j_ is the effect of sex (fixed), γ_k_ is the effect of block (random), and the ε_ijk_ is the residual error.

A planned contrast comparing the MI-injected groups (MI 12 + MI 24) with the no-MI groups (PC + NC) was specified using the ESTIMATE statement in the MIXED procedure.

Hatchability data were analyzed using the Chi-squared test, with each egg treated as an individual observation (hatched = 1 and unhatched = 0) to compare proportions of hatched chicks among treatment groups.

Metabolite profile visualization and multivariate analyses were performed using MetaboAnalyst 6.0 (Pang et al., 2024). After log transformation and Pareto scaling, a combined dataset containing all 145 measurable metabolites of the IDQ p180 panel, MI-related variables, and individual body weight at slaughter was analyzed. Partial least-squares discriminant analysis (PLS-DA) was used as an exploratory approach to assess the overall multivariate structure, and model validity was evaluated using cross-validation and permutation tests. Variable importance in projection (VIP) scores (>1) were reported to indicate metabolites contributing most to the multivariate model; however, statistical inference relied exclusively on the SAS mixed-model analyses described above.

Variance homogeneity and normal distribution were verified for each trait. The results are presented as least-square means (LSmeans) and standard error of the mean (SEM). Percentage data were arcsine square root transformed before analysis to stabilize variances; however, LSmeans (±SEM) are presented as back-transformed percentages for clarity.

Differences were considered statistically significant at *P* < 0.050, and trends were accepted at 0.050 ≤ P < 0.100.

## 3 Results

### 3.1 Hatchability and performance traits

Hatchability rates were 92.7% ± 2.5% (MI 12), 90.9% ± 2.8% (MI 24), 89.1% ± 3.0% (PC), and 97.3% ± 1.6% (NC), without significant differences among groups (df = 3; χ^2^ = 5.86; *P* = 0.118).

#### 3.1.1 Overall treatment effects

Body weight differed among treatments at d 0, 21, 28, and 35 (*P* < 0.050), with PC consistently showing the highest values (Table 1). ADG was numerically lower in MI 12 and MI 24 groups during d 7–28 (*P* < 0.100) and over the entire experimental period (d 0–35, *P* = 0.068). ADFI differed at d 7–14 (*P* = 0.016), with MI 24 consuming less feed than PC and NC. FCR and SR were not significantly affected by treatment, although a minor trend for higher SR in MI 12 and MI 24 groups was observed during d 0–21 (*P* = 0.069).

**TABLE 1:** Effect of *in ovo* treatment on performance traits in broiler chickens from d 0 to d 35.

Parameter/period (d)	MI 12^1^	MI 24^2^	PC^3^	NC^4^	*SEM*	*P*-value	
						Treatment	MI^5^ vs. no-MI
BW, g							
d 0	44.0^ab^	44.2^b^	44.9^c^	43.8^a^	0.14	<0.001	0.180
d 7	213.3	211.6	216.8	213.9	2.46	0.583	0.282
d 14	581.1	564.1	589.2	586.8	7.42	0.096	0.047
d 21	1172^ab^	1146^a^	1207^b^	1197^b^	15.4	0.042	0.001
d 28	1968^ab^	1929^a^	2029^b^	2018^b^	25.8	0.036	0.007
d 35	2823^a^	2792^a^	2918^b^	2878^ab^	27.8	0.036	0.007
ADG, g/d							
d 0–7	24.2	23.8	24.5	24.3	0.38	0.634	0.292
d 7–14	52.6	50.4	53.0	53.3	0.82	0.068	0.048
d 14–21	84.4	83.2	88.4	86.9	1.50	0.081	0.016
d 21–28	113.8	111.1	117.4	117.4	2.08	0.087	0.018
d 28–35	122.1	122.3	125.0	122.9	2.71	0.781	0.456
d 0–35	79.3	77.8	81.0	80.3	0.86	0.068	0.020
ADFI, g/d							
d 0–7	25.4	25.6	25.6	25.1	0.28	0.562	0.634
d 7–14	59.5^ab^	58.2^a^	60.6^b^	59.9^b^	0.62	0.016	0.009
d 14–21	106.2	104.7	110.3	108.3	1.59	0.103	0.024
d 21–28	154.3	150.7	156.4	155.0	2.01	0.195	0.078
d 28–35	179.7	177.1	182.2	182.5	2.00	0.164	0.043
d 0–35	104.8	102.8	105.9	105.4	0.93	0.113	0.058
FCR, g/d							
d 0–7	1.05	1.08	1.04	1.03	0.02	0.191	0.091
d 7–14	1.13	1.16	1.14	1.12	0.01	0.263	0.370
d 14–21	1.26	1.26	1.25	1.25	0.01	0.521	0.144
d 21–28	1.36	1.36	1.33	1.33	0.01	0.105	0.016
d 28–35	1.47	1.45	1.45	1.49	0.02	0.535	0.646
d 0–35	1.32	1.32	1.31	1.31	0.01	0.445	0.112
SR, %							
d 0–7	100.0	99.9	99.9	100.0	0.07	0.580	1.000
d 0–14	100.0	99.9	99.2	100.0	0.12	0.248	0.457
d 0–21	100.0	99.9	97.4	99.5	0.19	0.069	0.028
d 0–28	99.9	99.5	97.4	97.9	0.26	0.239	0.053
d 0–35	99.9	99.2	96.9	97.9	0.32	0.283	0.079

Data are given as LSmeans ± SEM, *n* = 8 animals per treatment group.

Abbreviations: BW, average body weight; ADG, average daily weight gain; ADFI, average daily feed intake; FCR, feed conversion rate; SR, survival rate.

^1^MI 12, *in ovo* injection of 12 μmol myo-inositol.

^2^MI 24, *in ovo* injection of 24 μmol myo-inositol.

^3^PC, positive control, *in ovo* injection of 0.9% saline.

^4^NC, negative control, non-injected.

^5^MI vs. no-MI, MI treatment (MI 12+MI 24) vs. controls (PC + NC).

^a,b,c^Different superscript letters within a row indicate statistically significant differences between treatment groups at *P* < 0.05.

#### 3.1.2 Planned contrast MI vs. no-MI

Planned contrasts indicated that MI-injected groups had lower BW than no-MI groups from d 14 onward and lower ADG during d 7–28 and d 0–35 (*P* < 0.050), consistent with the ADG trends observed in overall treatment effects. Contrast analysis confirmed lower ADFI in MI-injected groups than in no-MI groups during d 7–21 and d 28–35 (*P* < 0.050), whereas FCR differed only during d 21–28 (*P* = 0.016), reflecting better feed efficiency in the no-MI groups. MI-injected groups had higher SR than no-MI groups during d 0–21 (*P* = 0.028) and showed a tendency for higher SR up to d 35 (*P* < 0.100).

At slaughter (day 35), individual BW did not differ significantly among treatment groups (*P* = 0.109; Table 2). However, a planned contrast revealed lower BW in MI-injected birds than in no-MI groups (*P* = 0.020). Sex significantly influenced BW, with males being heavier than females (2939 ± 32.2 g vs. 2774 ± 26.0 g; *P* = 0.008).

**TABLE 2:** Effect of *in ovo* treatment on body weight, myo-inositol concentration, and related enzyme expression in plasma and tissues of broiler chickens at d 35.

Parameter	MI 12^1^	MI 24^2^	PC^3^	NC^4^	Female	Male	Pooled SEM	*P*-value		
								Treatment	Sex	MI^5^ vs. no-MI
Body weight, g	2798	2815	2913	2899	2774^x^	2939^y^	39.24	0.109	0.008	0.020
Plasma MI, mmol/L	0.59	0.60	0.60	0.51	0.67^x^	0.48^y^	0.05	0.623	0.001	0.484
Liver MI, mg/g protein	22.5	28.0	32.6	30.7	28.5	28.4	4.9	0.488	0.994	0.231
Muscle MI, mg/g protein	13.7	15.0	13.9	11.4	13.5	13.5	1.4	0.458	0.968	0.282
Kidney MI, mg/g protein	15.9	16.5	16.8	14.1	14.2	17.5	2.4	0.871	0.367	0.797
Liver IMPase 1, pg/mg protein	0.79	0.78	1.02	0.85	0.82	0.90	0.08	0.174	0.424	0.104
Muscle IMPase 1, pg/mg protein	1.74	2.25	2.02	2.78	2.26	2.14	0.38	0.099	0.760	0.204
Kidney MIOX, pg/mg protein	0.58	0.71	0.60	0.65	0.65	0.62	0.05	0.343	0.736	0.705

Data are given as LSmeans ± SEM, *n* = 8 animals per group, female n = 19 and male n = 13.

^1^MI 12, *in ovo* injection of 12 μmol myo-inositol.

^2^MI 24, *in ovo* injection of 24 μmol myo-inositol.

^3^PC, positive control, *in ovo* injection of 0.9% saline.

^4^NC, negative control, non-injected.

^5^MI vs. no-MI, MI treatment (MI 12+MI 24) vs. controls (PC + NC).

^a,b,c^Different superscript letters within a row indicate statistically significant differences between treatment groups and between sexes (^*x, y*^) at *P* < 0.05.

### 3.2 MI concentration and IMPase 1 and MIOX expressions

The MI concentration in fresh non-incubated eggs averaged 2.98 ± 0.18 μmol/g DM in albumen and 2.06 ± 0.18 μmol/g DM in yolk, corresponding to 14.5 ± 1.66 and 23.5 ± 1.66 µmol per fraction, respectively.

MI concentrations in plasma and tissues, along with the expression of related enzymes, did not differ among treatment groups (Table 2). Sex effects were observed only in plasma MI concentration, with higher plasma MI in females than in males (*P* < 0.001). No other tissue MI concentrations or enzyme expressions differed by sex.

### 3.3 Plasma metabolite profiles

**FIGURE 1:**
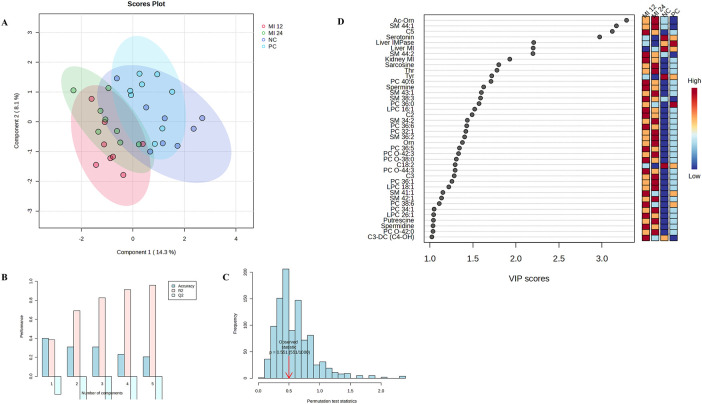
**(A)** Two-dimensional partial least-squares discriminant analysis scores plot for the four *in ovo* treatment groups. **(B)** Cross-validation results. **(C)** Permutation test. **(D)** Variable importance in projection (VIP) scores identifying metabolites contributing most to group differentiation. MI 12, *in ovo* injection of 12 μmol myo-inositol; MI 24, *in ovo* injection of 24 μmol myo-inositol; PC, positive control, *in ovo* injection of 0.9% saline; NC, negative control, non-injected.

**FIGURE 2:**
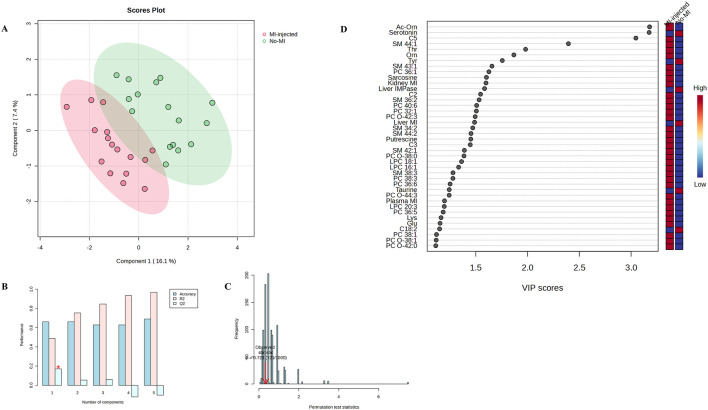
**(A)** Two-dimensional partial least-squares discriminant analysis scores plot comparing MI-injected (MI 12 + MI 24) with no-MI (PC + NC) groups. **(B)** Cross-validation results; the asterisk represents the maximum quality assessment statistic (Q^2^). **(C)** Permutation test. **(D)** Variable importance in projection (VIP) scores identifying metabolites contributing most to group differentiation.

**FIGURE 3:**
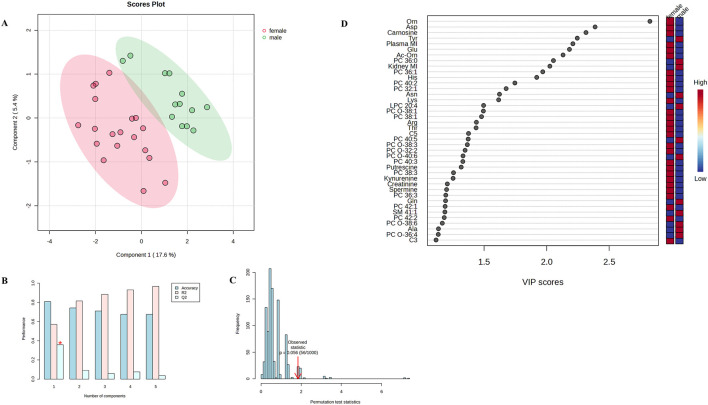
**(A)** Two-dimensional partial least-squares discriminant analysis scores plot illustrating sex-related differences in plasma metabolite profiles. **(B)** Cross-validation results; the asterisk represents the maximum quality assessment statistic (Q^2^). **(C)** Permutation test. **(D)** Variable importance in projection (VIP) scores identifying metabolites contributing most to group differentiation.

Exploratory PLS-DA was first performed to assess multivariate structure of the metabolite data (Figures 1–3), whereas statistical inference relied exclusively on univariate mixed-model analyses. Metabolites significantly affected by treatment or by the MI vs. no-MI contrast are presented in Table 3, those affected by sex are presented in Table 4, and the complete dataset is provided in Supplementary Table S1.

**TABLE 3:** Effect of *in ovo* treatment on differential metabolite concentrations and derived metabolic ratios in plasma of broiler chickens at d 35 (µmol/L).

Parameter	MI 12^1^	MI 24^2^	PC^3^	NC^4^	Female	Male	Pooled SEM	*P*-value		
								Treatment	Sex	MI^5^ vs. no-MI
Acylcarnitines										
Acetylcarnitine (C2)	0.84	0.81	0.76	0.63	0.76	0.77	0.055	0.087	0.920	0.044
Nonanoylcarnitine (C9)	0.025^a^	0.022^ab^	0.019^bc^	0.015^c^	0.017^x^	0.023^y^	0.002	0.002	0.010	0.001
Amino acids										
Threonine	383.0	379.9	332.0	301.9	377.8	320.6	26.4	0.172	0.089	0.038
Biogenic amines										
Sarcosine	20.9	20.8	18.3	17.8	19.5	19.3	1.0	0.117	0.864	0.019
Serotonin	0.10	−0.19	0.57	1.25	0.71	0.16	0.42	0.113	0.301	0.030
Serotonin/Trp	0.001	−0.002	0.006	0.014	0.008	0.002	0.005	0.106	0.285	0.031
Spermine/spermidine	1.79^a^	1.31^b^	1.53^ab^	1.67^a^	1.71^x^	1.44^y^	0.11	0.026	0.045	0.614
Glycerophospholipids										
PC 30:2	0.08^ab^	0.09^ab^	0.10^a^	0.06^b^	0.08	0.08	0.009	0.027	0.579	0.681
PC 36:5	12.1^a^	11.4^ab^	10.9^ab^	9.8^b^	10.9	11.2	0.51	0.044	0.599	0.023
PC 36:6	0.42^a^	0.39^ab^	0.38^ab^	0.34^b^	0.38	0.39	0.019	0.023	0.816	0.020
PC 38:6	32.5	31.8	29.5	26.4	27.5^x^	32.6^y^	1.62	0.071	0.021	0.023
PC 40:6	18.4^ab^	19.1^a^	16.0^bc^	14.9^c^	15.8^x^	18.4^y^	0.84	0.016	0.018	0.002
PC O-38:0	1.85^a^	1.82^a^	1.69^ab^	1.48^b^	1.69	1.73	0.10	0.034	0.718	0.013
PC O-42:3	0.23	0.24	0.21	0.19	0.22	0.22	0.01	0.139	0.969	0.029
PC O-44:3	0.029	0.028	0.027	0.024	0.027	0.027	0.001	0.076	0.930	0.039
Sphingolipids										
SM 34:1	159.9^a^	157.1^ab^	158.0^a^	141.5^b^	151.6	156.6	4.63	0.047	0.416	0.097
SM 34:2	1.04^a^	1.01^a^	0.96^ab^	0.90^b^	1.01	0.94	0.03	0.049	0.133	0.014
SM 36:2	1.60	1.57	1.48	1.32	1.53	1.45	0.07	0.085	0.388	0.030
SM 38:3	0.33^a^	0.34^a^	0.29^b^	0.29^b^	0.30	0.33	0.01	0.033	0.088	0.004
SM 41:1	3.10^a^	2.92^a^	2.75^ab^	2.37^b^	2.53^x^	3.04^y^	0.14	0.007	0.007	0.004
SM 43:1	0.65^ab^	0.68^a^	0.59^bc^	0.54^c^	0.59	0.63	0.02	0.002	0.148	0.000
SM 44:2	0.14	0.13	0.09	0.10	0.10	0.13	0.02	0.177	0.237	0.036
OCFA-SMs/ECFA-SMs	0.030	0.030	0.029	0.028	0.028^x^	0.030^y^	0.001	0.119	0.001	0.031
Sum of ECFA-SMs	212.7^a^	206.3^ab^	207.1^a^	186.2^b^	201.5	204.6	6.53	0.043	0.72	0.074
Sum of LCFA-SMs	186.3^a^	181.0^ab^	182.5^a^	163.5^b^	176.2	180.4	5.67	0.039	0.573	0.085
Sum of OCFA-SMs	6.41^a^	6.06^a^	5.94^a^	5.23^b^	5.64	6.19	0.25	0.013	0.081	0.015
Sum of SMs	219.4^a^	212.6^a^	213.3^ab^	191.6^b^	207.4	211.1	6.75	0.041	0.676	0.069

Data are given as LS means ± SEM, *n* = 8 animals per group, female n = 19 and male n = 13.

Abbreviations: OCFA-SMs, odd-chain fatty-acid sphingomyelins; ECFA-SMs, even-chain fatty-acid sphingomyelins; LCFA-SMs, long-chain fatty-acid sphingomyelins; SMs, sphingomyelins.

^1^MI 12, *in ovo* injection of 12 μmol myo-inositol.

^2^MI 24, *in ovo* injection of 24 μmol myo-inositol.

^3^PC, positive control, *in ovo* injection of 0.9% saline.

^4^NC, negative control, non-injected.

^5^MI vs. no-MI, MI treatment (MI 12+MI 24) vs. controls (PC + NC).

^a,b,c^Different superscript letters within a row indicate statistically significant differences between treatment groups and between sexes (^*x, y*^) at *P* < 0.05.

**TABLE 4:** Effect of *in ovo* treatment on differential metabolite concentrations and derived metabolic ratios in plasma of broiler chickens at d 35 affected exclusively by sex (µmol/L).

Parameter	MI 12^1^	MI 24^2^	PC^3^	NC^4^	Female	Male	Pooled SEM	*P*-value		
								Treatment	Sex	MI^5^ vs. no-MI
Acylcarnitines										
C3-DC (C4-OH)	0.088	0.089	0.078	0.082	0.078^x^	0.091^y^	0.005	0.388	0.046	0.111
Amino acids										
Alanine	980.1	972.1	921.6	917.3	878.6^x^	1017.0^y^	49.0	0.771	0.029	0.311
Arginine	534.4	547.4	553.4	569.3	600.5^x^	501.5^y^	30.3	0.877	0.017	0.530
Asparagine	274.0	278.3	270.6	259.9	244.4^x^	297.0^y^	10.2	0.715	0.000	0.349
Aspartic acid	37.5	48.9	46.1	36.7	48.9^x^	35.7^y^	4.67	0.224	0.032	0.712
Glutamine	1473	1532	1530	1487	1397^x^	1614^y^	65.0	0.853	0.009	0.918
Glutamic acid	180.0	210.2	186.6	179.1	213.4^x^	164.5^y^	15.4	0.546	0.017	0.433
Histidine	78.4	82.6	84.8	72.5	90.1^x^	69.0^y^	6.51	0.634	0.014	0.802
Ornithine	45.8	49.0	39.6	38.1	53.5^x^	32.7^y^	6.41	0.697	0.014	0.246
Phenylalanine	139.3	145.8	142.5	148.3	138.1^x^	149.7^y^	5.10	0.419	0.046	0.501
Tyrosine	234.6	252.3	273.9	280.1	223.2^x^	297.3^y^	23.0	0.364	0.001	0.125
Fisher ratio^5^	1.39	1.41	1.22	1.24	1.47^x^	1.16^y^	0.09	0.473	0.011	0.123
Non ess/ess AAs^6^	3.20	3.02	3.25	3.30	3.00^x^	3.38^y^	0.13	0.605	0.023	0.261
Sum of AAAs^7^	466.9	489.8	506.3	516.6	453.9^x^	535.9^y^	25.8	0.379	0.008	0.156
Biogenic amines										
Carnosine	22.9	23.3	25.4	20.2	26.5^x^	19.3^y^	1.62	0.224	0.001	0.864
Creatinine	2.90	2.84	2.94	2.79	3.07^x^	2.66^y^	0.15	0.923	0.038	0.964
ADMA/Arg^8^	0.002	0.002	0.002	0.002	0.002^x^	0.003^y^	0.0001	0.942	0.001	0.882
Glycerophospholipids										
PC 28:1	0.26	0.24	0.28	0.25	0.27^x^	0.25^y^	0.01	0.083	0.019	0.151
PC 36:1	126.6	138.6	119.0	110.5	138.5^x^	108.8^y^	11.6	0.525	0.047	0.181
PC 36:4	160.8	156.5	156.1	144.0	145.3^x^	163.4^y^	5.77	0.233	0.023	0.177
PC 40:2	0.57	0.61	0.60	0.58	0.65^x^	0.53^y^	0.03	0.769	0.004	0.946
PC 40:5	20.1	20.7	19.8	18.9	18.0^x^	21.7^y^	0.90	0.667	0.002	0.287
PC O-32:2	0.35	0.32	0.34	0.33	0.36^x^	0.31^y^	0.01	0.430	0.002	0.819
PC O-36:3	4.45	4.19	4.46	4.10	4.55^x^	4.05^y^	0.15	0.289	0.012	0.827
PC O-36:4	16.9	16.6	16.9	15.8	15.5^x^	17.5^y^	0.63	0.579	0.024	0.563
PC O-38:3	1.71	1.70	1.68	1.58	1.78^x^	1.56^y^	0.07	0.611	0.018	0.322
PC O-38:6	2.18	2.14	2.11	1.97	1.96^x^	2.24^y^	0.09	0.358	0.016	0.201
PC O-40:6	1.74	1.71	1.71	1.57	1.55^x^	1.81^y^	0.08	0.465	0.022	0.357
Sphingolipids										
SM 40:4	0.25	0.27	0.25	0.21	0.22^x^	0.26^y^	0.02	0.123	0.040	0.069

Data are given as LSmeans ± SEM, *n* = 8 animals per group, female n = 19 and male n = 13.

^1^MI 12, *in ovo* injection of 12 μmol myo-inositol;

^2^MI 24, *in ovo* injection of 24 μmol myo-inositol;

^3^PC, positive control, *in ovo* injection of 0.9% saline;

^4^NC, negative control, non-injected;

^5^Fisher ratio, the ratio of branched-chain amino acids to aromatic amino acids;

^6^Non ess/ess AAs, the fraction of nonessential amino acids relative to essential amino acids;

^7^Sum of AAAs, sum of aromatic amino acids;

^8^ADMA/Arg, the fraction of asymmetrically dimethylated arginine (ADMA) relative to the unmodified arginine pool.

^x, y^Different superscript letters within a row indicate statistically significant differences between sexes at P < 0.05.

#### 3.3.1 Main effect of *in ovo* treatment

PLS-DA analysis for the treatment effect showed weak discrimination (Figure 1A), as supported by cross-validation (maximum Q^2^ = −0.256; Figure 1B) and permutation tests (*P* = 0.551; Figure 1C). VIP scoring identified 40 metabolites with VIP >1 (Figure 1D), representing variables that contributed most to the tentative discrimination pattern. Univariate mixed-model analyses (Table 3) revealed significant treatment effects for C9 (*P* = 0.002), the spermine/spermidine ratio (*P* = 0.026), multiple glycerophospholipid species (PC 30:2, PC 36:5, PC 36:6, PC 40:6, and PC O-38:0), and sphingomyelins (SM 34:1, SM 34:2, SM 38:3, SM 41:1, and SM 43:1) (*P* < 0.050). Additionally, total even-chain fatty-acid sphingomyelins (ECFA-SMs), long-chain fatty-acid sphingomyelins (LCFA-SMs), odd-chain fatty-acid sphingomyelins (OCFA-SMs), and total sphingomyelins were elevated in all injected groups (*P* < 0.050).

#### 3.3.2 Contrast analysis: MI vs. no-MI

When MI-injected (MI 12 + MI 24) and no-MI (PC + NC) groups were compared directly, PLS-DA again revealed limited discrimination (Figure 2A), supported by low Q^2^ in cross-validation (maximum Q^2^ = 0.127; Figure 2B) and weak permutation performance (*P* = 0.721; Figure 2C). The 40 metabolites with VIP >1 (Figure 2D) were the main contributors to this pattern, and univariate analyses confirmed significant differences for several metabolites (Table 3). Concentrations of acylcarnitines C2 (*P* = 0.044) and C9 (*P* = 0.001), threonine (*P* = 0.038), and sarcosine (*P* = 0.019) were higher in MI-injected birds. In contrast, serotonin (*P* = 0.030) and the serotonin-to-tryptophan ratio (*P* = 0.031) were lower. Increased concentrations of several glycerophospholipids (PC 36:5, PC 36:6, PC 38:6, PC 40:6, PC O-38:0, PC O-42:3, and PC O-44:3; *P* < 0.050) and sphingomyelins (SM 34:2, SM 36:2, SM 38:3, SM 41:1, SM 43:1, and SM 44:2; *P* < 0.050), along with higher sum of OCFA-SMs and the OCFA-SMs/ECFA-SMs ratio (*P* < 0.050), were observed in MI-injected groups.

#### 3.3.3 Main effect of sex

PLS-DA examining sex differences showed moderate visual separation between males and females (Figure 3A). However, cross-validation indicated low predictive performance (maximum Q^2^ = 0.358; Figure 3B), and permutation tests provided only borderline support for model stability (*P* = 0.056; Figure 3C). VIP scoring identified the variables contributing most to the observed structure (Figure 3D). The metabolites and metabolic ratios exhibiting significant sex effects were identified through univariate mixed-model analyses and are summarized in Table 4. Among acylcarnitines, plasma C3-DC (C4-OH) was higher in males than in females (*P* = 0.046). Male birds had higher concentrations of alanine, asparagine, glutamine, phenylalanine, and tyrosine and sum of aromatic AAs (AAAs), whereas female birds exhibited higher levels of arginine, aspartic acid, glutamic acid, histidine, and ornithine. Male birds showed a higher nonessential-to-essential AA ratio and sum of AAAs, whereas female birds exhibited a higher Fisher ratio. In addition, female birds had higher carnosine (*P* = 0.001) and creatinine (*P* = 0.038) concentrations, whereas male birds showed a higher asymmetric dimethylarginine-to-arginine ratio (ADMA/Arg). Female individuals had higher PC 28:1, PC 36:1, PC 40:2, PC O-32:2, PC O-36:3, and PC O-38:3, whereas male individuals exhibited higher PC 36:4, PC 40:5, PC O-36:4, and PC O-38:6. Some sphingolipids were affected by both treatment and sex, but only SM 40:4 showed a significant effect of sex alone, with higher concentrations in male individuals (*P* = 0.040).

## 4 Discussion

Myo-inositol plays essential roles in phosphoinositide signaling, membrane dynamics, antioxidant responses, and cellular energy metabolism (Gonzalez-Uarquin et al., 2020c). Beyond its established metabolic roles, MI was associated with metabolic programming (Nissen et al., 2011), intestinal function, and phosphate absorption across species (Ogunribido et al., 2022; Shomina et al., 2026), prompting the hypothesis that a transient elevation in MI before hatch might induce regulatory adjustments affecting the postnatal metabolic phenotype in broilers. To evaluate this, MI was administered *in ovo* into the amniotic fluid of developing embryos. Relative to the average MI content of the egg albumen (Sommerfeld et al., 2020), *in ovo* injections supplied approximately 0.8 (MI 12) and 1.7 (MI 24) times the MI naturally present in this fraction. When scaled to embryo body weight, these doses fall within the range of daily MI exposure typically achieved in adult birds fed diets supplemented with approximately 0.05%–0.15% MI (Sommerfeld et al., 2025). After 35 days of the grow-out period, one bird per pen was selected for sampling based on the proximity of its body weight to the mean pen weight, which inadvertently resulted in unequal sex ratios across treatments: MI 12 and PC had four male and four female birds each, NC had five male and three female birds, and MI 24 consisted solely of female birds. To account for this imbalance and its potential impact on individual-level traits, sex was included as a fixed factor in all corresponding statistical analyses. Nevertheless, the absence of male individuals in MI 24 precluded detection of sex-by-treatment interactions, indicating that some MI-associated metabolic responses cannot be interpreted entirely independently of sex. Although this statistical approach provided a conservative correction, the imbalance in sex distribution remains a limitation of the study.

### 4.1 Effect of MI *in ovo* injection on broiler performance and metabolism

The amniotic cavity is an established and widely used route for *in ovo* nutrient delivery, allowing the embryo to ingest substances through the gastrointestinal and respiratory systems during the final days of incubation (Jochemsen and Jeurissen, 2002; Das et al., 2021). However, its involvement in osmotic regulation raised concerns about solute concentrations. Electrolytes and solutes introduced into the amnion can potentially disrupt established osmotic gradients essential for water influx into embryonic tissues (McGruder et al., 2011). According to Uni and Ferket (2003), the critical osmolarity threshold for *in ovo* solutions is 500–600 mOsm/L, above which embryonic viability may be compromised. Importantly, the osmolarity of the solutions used in our trial remained well below this threshold (308–330 mOsm/L), supporting the safety and physiological compatibility of the applied doses. In this study, hatchability remained within acceptable commercial ranges across groups, indicating that the applied MI solutions were generally well tolerated. However, it must be acknowledged that injected groups experienced additional procedural steps (shell puncturing, fluid injection, and wax sealing) that were not applied to the non-injected NC group. Even when physiologically inert solutions were used, *in ovo* manipulation *per se* slightly depressed hatchability, depending on the timing, injection site, and injected volume (McGruder et al., 2011; Pawłowska et al., 2022). Thus, minor numerical reductions in hatchability in injected groups likely reflect the embryo’s sensitivity to *in ovo* manipulation. This interpretation is further supported by the absence of adverse effects on body weight at hatch: chicks from the MI 24 and PC groups showed higher body weight than those in the NC group. Although MI might have conferred metabolic benefits, such as enhancing insulin sensitivity and promoting glucose uptake (Croze and Soulage, 2013), the body weight differences observed at hatch in the present study might reflect normal variation associated with *in ovo* handling and embryo responsiveness.

Analysis of plasma and tissue MI concentrations and the expression of MI-related enzymes revealed no differences among treatment groups at slaughter, indicating that *in ovo* MI administration did not induce long-term systemic alterations in MI metabolism. The absence of treatment effects on tissue MI concentrations, hepatic and muscle IMPase 1 expression, and renal MIOX expression likely reflected the strong homeostatic control of MI metabolism in broilers, whereas transient elevations in MI availability were buffered and normalized during post-hatch development. Similar observations were reported in dietary phytase studies in broilers, where increases in luminal or circulating MI did not consistently translate into changes in hepatic or renal MI concentrations (Gonzalez-Uarquin et al., 2020b; Sprigg et al., 2022) or in IMPase 1 expression in the liver or MIOX expression in the kidney (Gonzalez-Uarquin et al., 2020b). Thus, MI turnover appeared to be tightly regulated, and moderate fluctuations in MI supply, whether through phytase-mediated release or short-term embryonic supplementation, did not lead to persistent alterations in its metabolism. The correlation patterns observed in the present study further support this interpretation: hepatic MI concentration showed a moderate positive association with IMPase 1 expression (Spearman r = 0.48; *P* = 0.005), suggesting coordinated regulation between MI availability and its regeneration capacity in the liver, consistent with previous studies in poultry (Gonzalez-Uarquin et al., 2021). Additionally, a trend toward a negative correlation was observed between plasma MI and liver IMPase 1 (Spearman r = −0.32; *P* = 0.073), indicating a subtle reciprocal relationship between circulating MI and its hepatic regeneration. Notably, Gonzalez-Uarquin et al. (2021) reported a positive association between plasma MI concentration and renal MIOX expression in laying hens, implying that MI degradation in the kidney may adjust to systemic MI availability. In the present study, MI concentrations in muscle and kidney, along with muscle IMPase 1 and kidney MIOX expression, were not correlated with plasma or tissue MI levels. These outcomes collectively indicate that MI homeostasis was effectively stabilized through endogenous regulatory mechanisms, preventing long-lasting effects of the embryonic MI exposure.

Despite unchanged plasma or tissue MI concentrations at slaughter, several notable effects on broiler performance and metabolism were detected. The reduced BW observed from day 14 onward in MI-injected groups suggested a lasting influence of embryonic MI exposure on growth dynamics. As the MI 24 group contained only female birds at sampling, despite mixed-sex pens, the overall reduced body weight in MI treatments may partly reflect sex-dependent variability in MI responsiveness. Interestingly, this reduction in growth was accompanied by a trend to improved survival during the post-hatch period, indicating a potential trade-off between the growth rate and resilience. A likely factor contributing to reduced growth was the lower feed intake observed in MI-injected groups, particularly between days 7 and 21, suggesting that MI may modulate energy metabolism or appetite regulation, although the underlying mechanisms remain unclear.

In the present study, MI exposure influenced peripheral serotonin metabolism as MI-injected birds showed lower plasma serotonin concentrations, along with a reduced serotonin-to-tryptophan ratio, than no-MI birds. In chickens, circulating serotonin is synthesized mainly by enterochromaffin cells of the intestinal mucosa and enters the bloodstream, where most of it becomes clustered within thrombocytes and other blood cells (Alberghina et al., 2020). Because gut-derived serotonin does not cross the blood–brain barrier and only a minor fraction is produced centrally, plasma serotonin reflects peripheral serotonergic activity. Tryptophan is the precursor for serotonin synthesis, and conversion efficiency depends on its availability and the activity of tryptophan hydroxylase (Alberghina et al., 2020). Accordingly, the reduced serotonin-to-tryptophan ratio in MI-injected birds may indicate lower peripheral serotonin synthesis or a redistribution of tryptophan toward alternative metabolic pathways. Peripheral serotonin levels are sensitive to physiological challenges; for example, elevated corticosterone during embryogenesis reduced whole-blood serotonin in young chickens (Ahmed et al., 2014). Peripheral serotonin has also been associated with inflammatory and metabolic states in poultry, with higher plasma serotonin observed in hens with footpad dermatitis (Alberghina et al., 2020) and in metabolic clusters characterized by pro-inflammatory features (Szentgyörgyi et al., 2025). In our study, MI-injected birds had lower plasma serotonin, which contrasted with findings from broilers receiving continuous dietary MI supplementation, where plasma serotonin and dopamine concentrations were increased (Gonzalez-Uarquin et al., 2020a). Beyond effects on serotonin metabolism, MI-injected birds exhibited higher concentrations of acylcarnitine C2 and C9, the metabolites commonly associated with mitochondrial β-oxidation and acetyl-CoA handling. C2 carnitine or acetylcarnitine is the main acylcarnitine found in the plasma. In addition to its important role in energy production, C2 provides acetyl groups for the synthesis of acetylcholine (Dambrova et al., 2022). Acetylcarnitine serves as a buffer for mitochondrial acetyl-CoA via the carnitine acetyltransferase reaction, which also interacts with the regulation of the pyruvate dehydrogenase complex and thereby contributes to substrate partitioning between carbohydrate and fatty-acid oxidation (Schooneman et al., 2016). Recent experimental work in avian fibroblasts demonstrated that MI can suppress pyruvate dehydrogenase activity and shift mitochondrial fuel utilization toward fatty acids, resulting in increased fatty-acid oxidation when substrates are available (Domer et al., 2025). The elevated C2 and C9 levels observed in MI-injected birds are compatible with such a shift in mitochondrial substrate use and suggest that MI exposure during late embryogenesis may have influenced post-hatch mitochondrial energy metabolism. MI-injected birds also exhibited higher plasma concentrations of phosphatidylcholines and sphingomyelins. These lipid classes participate in membrane turnover and can contribute to diacylglycerol (DAG)-related signaling pathways through the activities of sphingomyelin synthase and phospholipase D (Antal and Newton, 2013; Scott et al., 2013). In particular, phospholipase D catalyzes the hydrolysis of phosphatidylcholine to generate phosphatidic acid, a lipid second messenger positioned at the intersection of multiple lipid metabolism and cell signaling pathways, including those regulating membrane trafficking, survival, and proliferation. Phosphatidic acid can be dephosphorylated to DAG (Scott et al., 2013). Sphingomyelin synthase synthesizes SM from ceramide and PC, generating DAG as a by-product (Taniguchi and Okazaki, 2014). This shift toward DAG-enriched membrane microdomains may favor protein kinase C recruitment and related signaling processes.

MI exposure also altered several amino-acid-related metabolites, including threonine and sarcosine. Threonine is an essential amino acid playing central roles in energy metabolism, protein synthesis, and mucin production, and it can be catabolized into acetyl-CoA, glycine, or pyruvate, depending on metabolic demand (Tang et al., 2021). The elevated threonine concentrations observed in MI-injected birds may, therefore, reflect adjustments in amino acid utilization associated with broader metabolic adaptation, for example, changes in mitochondrial β-oxidation and acetyl-CoA flux that reduce threonine catabolism or shift its use toward biosynthetic pathways. Sarcosine is produced from glycine via glycine-N-methyltransferase or from dimethylglycine via dimethylglycine dehydrogenase, and it can be reconverted to glycine through sarcosine dehydrogenase or pipecolic acid oxidase. These reactions are associated with the methionine cycle, where methyl groups contribute to S-adenosylmethionine formation and support a wide range of transmethylation reactions (Strmiska et al., 2019). Thus, the elevated sarcosine observed in MI-injected birds may suggest a shift within one-carbon and methyl-donor metabolism.

The limited but coherent metabolite differences observed in MI-injected birds, including altered serotonin levels and changes in acylcarnitines, phospholipids, threonine, and sarcosine, indicate that MI exposure induced a subtle metabolic signature detectable at slaughter age. Given the high interindividual variability and modest effect sizes, these findings should be interpreted as hypothesis-generating, suggesting that MI may influence specific aspects of post-hatch metabolic regulation. Whether such MI-related adaptations confer benefits or impose constraints when birds face increased metabolic or physiological demand remains to be determined.

### 4.2 Sex-related variations and their influence on treatment outcomes

Sex emerged as an important biological variable in our dataset and was considered in data interpretation and statistical analysis. Consistent with prior research, male broilers displayed higher BW and feed intake than female broilers at the same age (López et al., 2011; Da Costa et al., 2017). In our trial, female birds had higher plasma MI concentrations than male birds, and, to our knowledge, this is the first report of sex-dependent variation in plasma MI in poultry species. This finding may be related to higher circulating estradiol levels in female than in male commercial broilers (Holst-Schumacher et al., 2010). In previous studies on laying hens conducted within the P-Fowl Research Unit (Sommerfeld et al., 2024; Qasir et al., 2025), plasma MI and estradiol were positively correlated (Spearman r = 0.29, *P* = 0.009; unpublished data). In humans, a positive correlation between estradiol and MI in follicular fluid was reported (Chiu et al., 2002). At the metabolic level, most sex-related differences in our trial were observed in AA metabolism, which is in line with previous reports showing sex-specific AA requirements (England et al., 2023). Transcriptomic evidence also indicated sex- and age-dependent differences in intestinal nutrient transporter expression in poultry species, suggesting dimorphic nutrient utilization early post-hatch (Kaminski and Wong, 2018), whereas gut microbiota composition differences may further contribute to divergent nutrient handling (Lumpkins et al., 2008; Lee et al., 2017). In our study, male birds showed a lower ratio of branched-chain AA to aromatic AA (Fisher ratio), a pattern associated with hepatic lipidosis in fattening turkeys (Middendorf et al., 2019). They also exhibited a higher ADMA/arginine ratio, indicating potential inhibition of nitric oxide synthase and reduced nitric oxide bioavailability, a hallmark of vascular inflammatory stress in mammals (Sydow and Münzel, 2003). These results suggest that male birds may experience a more “tense” metabolic state, possibly reflecting higher anabolic pressure or subclinical inflammatory activation. However, it must be emphasized that these interpretations are based on data from mammals as comparable mechanistic evidence is largely lacking in poultry. Conversely, female birds had higher plasma carnosine concentrations (26.5 ± 1.2 vs. 19.3 ± 1.5 μmol/L; P = 0.001), suggesting stronger antioxidant buffering capacity that may limit oxidative and inflammatory propagation. Carnosine (β-alanyl-L-histidine) is a dipeptide with well-documented antioxidative and anti-aging properties (Hipkiss and Brownson, 2000). Changes were also observed in lipid metabolite classes, particularly phosphatidylcholines (PCs). Female birds had higher PC 28:1, PC 36:1, PC 40:2, PC O-32:2, PC O-36:3, and PC O-38:3, whereas male birds exhibited higher PC 36:4, PC 40:5, PC O-36:4, and PC O-38:6. However, interpretation of these findings remains challenging due to limited knowledge about the functional relevance of these lipid subclasses in chickens.

## 5 Conclusion

Transient myo-inositol exposure during embryogenesis was associated with modest but consistent differences in broiler growth dynamics and circulating metabolite profiles that remained detectable at slaughter age. Although overall multivariate discrimination between treatment groups was weak, univariate analyses revealed coordinated changes in metabolites related to mitochondrial functioning, membrane lipid composition, amino-acid metabolism, and peripheral serotonin metabolism, indicating that embryonic MI exposure can influence certain aspects of post-hatch metabolic regulation. Sex accounted for a substantial proportion of metabolic variation, with distinct sex-specific responses characterized by higher pro-inflammatory-associated markers in male birds and enhanced antioxidant-related capacity in female birds. However, the unbalanced sex distribution among MI-injected birds limited evaluation of potential sex-dependent MI responsiveness. Future studies are needed to establish the functional relevance of MI-induced metabolic reprogramming in both sexes.

## Data Availability

The original contributions presented in the study are included in the article/Supplementary Material; further inquiries can be directed to the corresponding author.
